# Patient acceptance of universal screening for hepatitis C virus infection

**DOI:** 10.1186/1471-2334-11-160

**Published:** 2011-06-06

**Authors:** Phillip O Coffin, Anne M Stevens, John D Scott, Joanne D Stekler, Matthew R Golden

**Affiliations:** 1 Division of Allergy and Infectious Diseases, University of Washington, Seattle WA, USA; 2 School of Medicine, University of Washington, Seattle WA, USA

## Abstract

**Background:**

In the United States, about 70% of 2.9-3.7 million people with hepatitis C (HCV) are unaware of their infection. Although universal screening might be a cost-effective way to identify infections, prevent morbidity, and reduce transmission, few efforts have been made to determine patient opinions about new approaches to screening.

**Methods:**

We surveyed 200 patients in August 2010 at five outpatient clinics of a major public urban medical center in Seattle, WA, with an 85.8% response rate.

**Results:**

The sample was 55.3% women, median 47 years of age, and 56.3% white and 32.7% African or African-American; 9.5% and 2.5% reported testing positive for HCV and HIV, respectively. The vast majority of patients supported universal screening for HCV. When presented with three options for screening, 48% preferred universal testing without being informed that they were being tested or provided with negative results, 37% preferred testing with the chance to "opt-out" of being tested and without being provided with negative results, and 15% preferred testing based on clinician judgment. Results were similar for HIV screening.

**Conclusions:**

Patients support universal screening for HCV, even if that screening involves testing without prior consent or the routine provision of negative test results. Current screening guidelines and procedures should be reconsidered in light of patient priorities.

## Background

Hepatitis C infection (HCV) is a neglected disease. Approximately 1.6% of the U.S. population (2.9 to 3.7 million people) is infected with HCV[1], 20-30% will progress to cirrhosis, and 49-75% are unaware of their infection[2]. HCV is the most common underlying cause of liver failure among persons undergoing liver transplant in the U.S. and kills approximately 14,000 Americans annually[2]. The number of new cases of cirrhosis from HCV is predicted to increase by over 30% as of 2020 and subsequent liver decompensation, hepatocellular carcinoma, and death will continue to increase into the 2030s[3]. Current therapy for HCV is curative for about 50% of treated patients and could decrease these complications by 16-42% if implemented broadly[3]. Soon to be available protease inhibitor regimens are expected to cure over 70%[4,5] and thus could have a more dramatic impact. Yet despite the scale of the problem and the availability of increasingly effective treatment, relatively little has been done to identify persons with HCV and ensure their treatment.

National guidelines recommend that testing for HCV be limited to persons with identified risk factors, such as injection drug use, a blood transfusion prior to 1992, or elevated liver function tests[6]. Although these recommendations are based on epidemiologic data and 1998 Centers for Disease Control and Prevention (CDC) guidelines[7], barriers to risk-based screening have resulted in inadequate detection of HCV. Most importantly, patients with a remote history of injection drug use, the primary risk factor for HCV, are often reluctant to admit that behavior to a clinician; only 21% of respondents with HCV reported IDU in the National Health and Nutrition Examination Survey IV [8]. In all but the most complex guidelines, risk factor-based screening also fails to account for suspected transmission routes, such as remote iatrogenic transmission, intranasal drug use[9] or even a fist fight[10]. Overall, risk factor based screening for HCV might be too complex for routine clinical care.

Due to barriers to risk-factor based HIV screening, including the ability to reliably assess risk in the clinical setting, the CDC in 2006 recommended routine "opt-out" general population screening for HIV[11]. The requirement for separate consent to test for HIV in many states and inadequate time for counseling[12] continue to impede testing, with attendant reductions in the proportion of patients screened for HIV[13]. Such barriers may still be too onerous to ensure detection of the estimated 21% of HIV-infected Americans who are unaware of their infection[14]. Likewise, another set of complex screening criteria or a clinician-driven approach to HCV screening may fail us in the effort to diagnose and care for the two to three million U.S. residents unaware of their HCV infection.

The debate surrounding patient consent for diagnostic testing or screening highlights the tension within the healthcare system between a desire to respect patient autonomy, contain costs, and maximize detection of treatable medical conditions. Unfortunately, patients are often the missing voice in this debate, and little is known about the relative importance patients place on being informed, consented, and tested. In considering a future HCV screening intervention, we evaluated patient opinion at a large public, urban hospital.

## Methods

We conducted an anonymous, self-administered, cross-sectional survey of outpatients at five clinics at Harborview Medical Center, a public urban hospital and regional trauma center in Seattle, WA, to define the importance that patients place on testing for HCV, compared to HIV and diabetes mellitus. We included HIV as testing requires special consent procedures, thus universal screening for HIV might raise additional concerns relative to HCV. We included diabetes because a manner of universal screening is already de facto present in every chemistry panel with a glucose result, but consent is not consistently obtained and normal results may be inconsistently discussed with patients (even abnormal results are only discussed with patients 66-88% of the time[15]). This study was approved by the Human Subjects Division of the University of Washington.

The instrument was piloted with five patients, met a Flesh-Kincaid fifth-grade reading level, and was in English, a language accessible to 90% of this hospital's patients (personal communication, Harborview Interpreter Services, 2 July 2010). We collected patient demographics and expectations as to physician communication regarding laboratory testing.

The remaining sections addressed HCV, HIV, and diabetes. Each section was preceded by a brief description of the disease and a statement that some people do not learn about their condition until they are sick. Initial questions in each section asked subjects' disease status and risk factors, followed by a series of questions about aspects of universal screening detailed in the accompanying tables. All questions were asked of HCV, but to ensure brevity only a subset of questions were included in sections on HIV and diabetes. The primary outcome variable, asked of HCV and HIV, allowed respondents to select one of three screening options: (1) universal screening of all blood samples without patient knowledge of the test and without receiving negative results, (2) universal screening without receiving negative results but with a chance to refuse testing, or (3) screening based on clinician judgment. Comments were solicited at the end of the survey.

Forty surveys were collected from each of five clinics (General Medicine, Family Medicine, Womens', General Surgery, and Orthopedics), selected to represent the diverse nature of outpatient care, including primary care serving a largely inner-city population and surgical care serving five northwest states. Researchers sequentially approached all patients in the waiting room after they had checked into the clinic and completed registration paperwork. Eligible subjects were more than 15 years of age, able to communicate in English, and had not previously completed the survey. Interested subjects were provided an "information sheet" in lieu of signed consent and, if they still wanted to participate, a survey and pencil. Subjects completed the survey in the waiting area or a clinic room and received $5 compensation when they returned the completed survey to the researcher. All data collection activities were completed in August 2010.

Data were analyzed with STATA 11.1, utilizing descriptive statistics, Pearson's Chi-square, and McNemar's test of matched pairs. To evaluate for any associations with the primary outcome of preferred screening method, we collapsed the two options for universal screening and compared those respondents to those who preferred screening by clinician judgment. Missing values were excluded. We coded optional comments based on identified themes.

## Results

A total of 233 patients were eligible and 200 (86%) completed the survey. Data were not collected for non-responders. Over half (55%) of respondents were women and the median age was 47 years (range 18-82). One-hundred twelve (56%) respondents were white, 65 (33%) were African or African-American, and 16 (8%) were Hispanic or Latino. Demographics compared to the 2010 census by clinic are presented in Table 1.

**Table 1:** Demographic characteristics of survey respondents (N = 200) and 2010 clinic census (N = 18,117)

	General Medicine	Family Medicine	Womens'	General Surgery	Orthopedics
	**Survey | Census**	**Survey | Census**	**Survey | Census**	**Survey | Census**	**Survey | Census**

Female	58% | 45%	45% | 59%	100%|100%	33% | 35%	41% | 36%

Median age (years)	40-49 | 50-59	40-49 | 40-49	20-29 | 30-39	40-49 | 40-49	40-49 | 40-49

Non-Hispanic White	53% | 47%	36% | 28%	23% | 31%	53% | 61%	65% | 76%

Non-Hispanic African-American	24% | 33%	48% | 44%	53% | 43%	20% | 20%	12% | 12%

Hispanic/Latino	5% | 7%	8% | 10%	8% | 15%	13% | 13%	8% | 5%

*Race/ethnicity was collected as two variables in the survey and one variable in the census (including Hispanic/Latino ethnicity), thus proportions do not match text

Twenty-three (12%) respondents reported ever injecting drugs, 39 (20%) reported having had a blood transfusion, and 10 (5%) were men who reported ever having sex with another man. A majority of participants reported previous testing for HIV, HCV and diabetes (see Table 2). Ninety-seven (50%) respondents reported that their doctor always told them about all of the tests being run on their blood and another 46 (24%) reported that happened most of the time; 174 (87%) reported that it was very important for them to know what tests were being done on their blood. The vast majority wanted to know if they had HCV, HIV, or diabetes. Most also indicated that the hospital should automatically test all patients for each condition, although analysis of matched pairs illustrated greater support for automatically testing all patients for HCV (p = 0.01) or HIV (p = 0.03) compared to diabetes.

**Table 2:** Opinions and preferences for screening for hepatitis C virus (HCV) compared to HIV and diabetes (N = 200)

	HCV	HIV	Diabetes
	No (%)	No (%)	No (%)
Have you been tested for ___?			

Yes	102 (51.0)	135 (68.2)	125 (63.5)

No/Don't Know	98 (49.0)	63 (31.8)	72 (36.5)

Tested positive for ___			

Yes	19 (18.8)	5 (3.7)	32 (25.6)

No/Don't Know	82 (81.2)	130 (96.3)	93 (74.4)

Would you want to know if you had ___?			

Yes	172 (86.9)	186 (94.9)	186 (95.4)

No/Don't Know	26 (13.1)	10 (5.1)	9 (4.6)

Should the hospital test ALL its patients for ___?			

Yes	149 (76.0)	142 (73.2)	131 (68.3)

No	16 (8.2)	23 (11.9)	37 (19.3)

Don't Know	31 (15.8)	29 (14.9)	24 (12.5)

One way to test everyone is to test all blood for ___ automatically. Would you be OK being tested this way?			

Yes	168 (86.2)	159 (80.7)	145 (75.9)

No	17 (8.7)	23 (11.7)	30 (15.7)

Don't Know	10 (5.1)	15 (7.6)	16 (8.4)

			

Preferred screening approach and potential concerns			

Which way is best to test for ___?			

Test all blood for ___. You might not know the test is being done. People who test negative would not be told the result.	91 (48.4)	91 (50.3)	n/a

Test all blood for ___. You would have a chance to ask NOT to be tested. People who test negative would not be told the result.	69 (36.7)	64 (35.4)	n/a

Let each doctor decide who to test.	28 (14.9)	26 (14.4)	n/a

Would it be OK to test you for ___ automatically without talking to you about the test first?			

Yes	67 (34.7)	69 (35.4)	n/a

No	120 (62.2)	112 (57.4)	n/a

Don't Know	6 (3.1)	14 (7.2)	n/a

If we found that you did NOT have ___, would it be okay if you did NOT get the results?			

Yes	43 (21.7)	n/a	77 (40.5)

No	148 (74.8)	n/a	108 (56.8)

Don't Know	7 (3.5)	n/a	5 (2.6)

Do you think it's better to be tested for ___ without knowing about it or not to be tested at all?			

Be tested without knowing	134 (75.3)	n/a	n/a

Not be tested at all	44 (24.7)	n/a	n/a

If we found out you DID have ___, would it be okay if someone from the health department told you the result? This would keep the result out of your medical record.			

Yes	143 (73.3)	n/a	n/a

No	28 (14.4)	n/a	n/a

Don't Know	24 (12.3)		

n/a = question was not asked for condition

Although almost two-thirds of respondents indicated that it would not be acceptable to test them for HCV without first telling them about the test, 75% indicated that they would prefer to be tested without knowing about it than not to be tested at all. More than three-quarters indicated that it would not be acceptable to be tested for HCV without receiving negative results. However, when presented with three screening options, the largest proportion favored universal screening without knowing about the test or receiving negative results and the smallest proportion favored relying on clinician judgment. Three-quarters of respondents indicated that they would accept being contacted with a positive result by a public health department representative.

There were no significant associations between demographic characteristics, risk factors, testing history or disease status and the acceptability of universal screening for HCV (see Table 3). Respondents who wanted to know if they were infected with HCV were more likely to support universal screening (p < 0.01). Acceptance of universal screening for HCV was also associated with agreement that the hospital should test all patients for HCV, and that testing should be automatic. Results of the analysis applied to HIV testing were the same as for HCV testing, including the absence of associations with demographics, risk factors, testing history or disease status, and positive associations with desire to know disease status and belief that testing should be automatic and for all patients (data not shown).

**Table 3:** Associations with hepatitis C virus screening preference (N = 188)

	Universal Screening No (row%)	Clinician Judgment No (row%)
Clinic		

General Medicine	32(82.1)	7(17.9)

Family Medicine	28(77.8)	8(22.2)

Womens'	36(90.0)	4(10.0)

General Surgery	30(85.7)	5(14.3)

Orthopedics	34(89.5)	4(10.5)

Gender		

Male	70(84.3)	13(15.7)

Female	89(85.6)	15(14.4)

Age (quartiles)		

16-32	44(89.8)	5(10.2)

33-47	49(86.0)	8(14.0)

48-56	31(79.5)	8(20.5)

57-82	35(83.3)	7(16.7)

Race/ethnicity		

White	87(83.7)	17(16.4)

African/African-American	52(85.3)	9(14.8)

Asian	8(88.9)	1(11.1)

Native Am/Alaskan Native	7(100.0)	0(0.0)

Hawaiian/Pacific Islander and Other	4(100.0)	0(0.0)

Hispanic/Latino		

No	143(84.1)	27(15.9)

Yes	14(93.3)	1(6.7)

How often does your doctor tell you ALL tests being done?		

Always/Most of the time	113(83.1)	23(16.9)

Sometimes/Never	44(89.8)	5(10.2)

Importance of knowing ALL tests being done		

Very	136(84.0)	26(16.0)

Somewhat/Not	24(92.3)	2(7.7)

Ever injected drugs not prescribed by a doctor		

Yes	17(81.0)	4(19.0)

No	142(85.5)	24(14.5)

History of blood transfusion		

Yes	36(92.3)	3(7.7)

No/Don't Know	124(83.2)	25(16.8)

Sexual orientation		

Heterosexual/straight	137(84.6)	25(15.4)

Gay/lesbian/queer	7(100.0)	0(0.0)

Bisexual	14(87.5)	2(7.4)

Have you been tested for HCV?		

Yes	82(87.2)	12(12.8)

No/Don't Know	78(83.0)	16(17.0)

Tested positive		

Yes	16(88.9)	2(11.1)

No/Don't Know	66(88.0)	9(12.0)

Would you want to know if you had HCV?*		

Yes	145(88.4)	19(11.6)

No/Don't Know	15(65.2)	8(34.8)

*p < 0.01

Themes identified on qualitative review of 85 optional comments included: testing would be good for individual patient health (29 comments), testing would be good for public health (20 comments), all patients should have to be tested to receive care (6 comments), patients should be told they are being tested (7 comments), testing should be done through patients' doctors (1 comment), cost of testing should be considered (3 comments), testing should be free (1 comment), enjoyed survey (19 comments), unrelated (3 comments). Two respondents suggested that universal screening should be limited to communicable diseases.

## Discussion

We found that most patients seeking outpatient care at an urban public hospital supported universal screening for HCV, as well as HIV and diabetes. While most preferred to know that they were being tested and to receive negative results, our findings suggest that patients believed that ensuring universal testing for HCV was more important than either soliciting patient consent for the test or providing negative test results. Notably, our results also suggest that patients were more supportive of universal screening for HCV and HIV than they were for diabetes, even though diabetes was the most common of the three medical conditions and diabetes screening is not governed by the sorts of regulations that limit HIV testing. Optional comments suggested that some patients might distinguish a greater public health value in patient awareness of infectious compared to non-communicable diseases.

We do not believe that these findings should immediately prompt health care providers or organizations to abandon efforts to inform patients and seek consent for testing. However, efforts to broadly screen populations for communicable diseases have met formidable barriers. Although the first studies documenting a high prevalence of undiagnosed HIV in some urban emergency departments were published almost 20 years ago[16], very few such departments have instituted expanded testing programs and most successful efforts have required hiring new staff, a cost that often makes the programs unsustainable. It remains uncertain whether medical institutions in the U.S. can widely screen patients for communicable diseases if such screening requires procedures that are not in place for other chronic conditions. While electronic medical record reminders have led to modest improvements in screening practices[17,18], more automated approaches or population-based public health interventions, possibly involving pooled ribonucleic acid screening,[19] may be necessary to address the burden of undiagnosed HCV. The results of this survey suggest that many patients would forego certain standards for consent to ensure timely diagnosis of HCV.

There are several limitations to this study. First and foremost, we are uncertain of the generalizability of our findings. We found no differences in screening preference between those seeking primary medical care and those seeking care for orthopedic and other surgical disease at this regional level 1 trauma center, and no differences based on HCV or HIV risk factors, testing history, or serostatus. Nonetheless, social and cultural differences may produce discordant results at other sites. Second, we did not confirm the self-reported histories of testing or disease status. To ensure brevity of the instrument, not all questions were asked of all conditions, limiting our ability to compare opinions across conditions. Finally, as a primarily quantitative instrument, we may have missed issues that could emerge in qualitative research, a concern we attempted to address through piloting the instrument and evaluating comments.

## Conclusions

In summary, our findings demonstrate that patients support universal testing for HCV, as well as HIV, that an "opt-out" program would be preferred if feasible, but that patients appear to place a higher priority on being tested than they do on the process of informed consent or the receipt of negative results. These findings should inform the priorities of clinicians, public health officials, and clinical risk managers.

## Competing interests

The authors declare that they have no competing interests.

## Authors' contributions

PC was responsible for protocol development, data collection, data analysis, and manuscript preparation. AS conducted data collection, entry and analysis and participated in manuscript preparation. JDS and JDS assisted with instrument development and manuscript preparation. MG conceived the study and assisted with design and manuscript preparation. All authors read and approved the final manuscript.

## Pre-publication history

The pre-publication history for this paper can be accessed here:

http://www.biomedcentral.com/1471-2334/11/160/prepub
